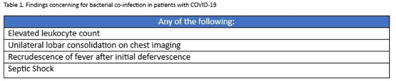# Assessment of antibiotic appropriateness in hospitalized veterans with COVID-19 in the VA MidSouth Healthcare Network (VISN9)

**DOI:** 10.1017/ash.2022.99

**Published:** 2022-05-16

**Authors:** Derek Forster, Morgan Johnson, Milner Staub, Jessica Bennett, Hans Scheerenberger, Angela Kaucher, Neena Thomas-Gosain, Kelly Davis

## Abstract

**Background:** Bacterial coinfections with COVID-19 appear to be rare, yet antibiotic use in this population is high. Limited guidance is available regarding the use of antibiotics in these patients. In response, a multidisciplinary group of physicians and pharmacists from 5 VISN9 facilities developed a guideline for the use of antibiotics with COVID-19 in July 2021. This guideline created a network-wide standard for antibiotic use and facilitates the assessment of antibiotic appropriateness in hospitalized veterans with COVID-19. **Methods:** In this observational, cross-sectional study, we reviewed veterans diagnosed with COVID-19 from August 1 through September 30, 2021, who were admitted to VISN9 facilities. Use of antibiotics was assessed during the first 4 days of admission. If antibiotics were prescribed, their use was determined to be appropriate or inappropriate based on the presence or absence of a finding concerning for bacterial coinfection as outlined in the guideline (Table 1). Additional data including procalcitonin results as well as positive sputum cultures were collected. **Results:** In total, 377 veterans were admitted for COVID-19 during the study period. Among them, 42 veterans (11%) received antibiotics for nonrespiratory infections and were removed from this analysis. Of the remaining 335 veterans, 229 (68%) received antibiotics and 116 (51%) of those met guideline criteria that were concerning for bacterial coinfection. Additionally, 32 (14%) of the 229 veterans who received antibiotics had >1 finding concerning for bacterial coinfection. Procalcitonin levels were obtained in 97 (42%) of 229. Only 33 veterans (14%) who received antibiotics had an elevated procalcitonin, and only 19 (8%) had a positive sputum culture. **Conclusions:** Antibiotic use was common in hospitalized veterans with COVID-19 in VISN9 facilities. This results are comparable to findings in the published literature. Among those receiving antibiotics early in their hospitalization, half were considered appropriate based on our guideline. Quality improvement initiatives are needed to improve implementation of the network guideline to reduce the overuse of antibiotics for management of COVID-19. Additionally, procalcitonin may be a helpful tool for hospitalized veterans with COVID-19.

**Funding:** None

**Disclosures:** None

**Table:**